# Neural substrates involved in anger induced by audio-visual film clips among patients with alcohol dependency

**DOI:** 10.1186/s40101-016-0102-x

**Published:** 2016-07-08

**Authors:** Mi-Sook Park, Bae Hwan Lee, Jin-Hun Sohn

**Affiliations:** Department of Counseling Psychology, Hanyoung Theological University, 290-42 Kyoungin-ro, Guro-gu, Seoul, 152-717 Republic of Korea; Department of Physiology, Brain Korea PLUS Project for Medical Science, Brain Research Institute, Yonsei University College of Medicine, 50-1 Yonsei-ro, Seodaemun-gu, Seoul, 03722 Republic of Korea; Department of Psychology, Chungnam National University, 99 Daehak-ro, Yuseong-gu, Daejeon, 305-764 Republic of Korea

**Keywords:** Alcohol dependency, Anger, fMRI, Audio-visual film clips

## Abstract

**Background:**

Very little is known about the neural circuitry underlying anger processing among alcoholics. The purpose of this study was to examine the altered brain activity of alcoholic individuals during transient anger emotion.

**Methods:**

Using functional magnetic resonance imaging (fMRI), 18 male patients diagnosed with alcohol dependence in an inpatient alcohol treatment facility and 16 social drinkers with similar demographics were scanned during the viewing of anger-provoking film clips.

**Results:**

While there was no significant difference in the level of experienced anger between alcohol-dependent patients and non-alcoholic controls, significantly greater activation was observed in the bilateral dorsal anterior cingulate cortex (dACC) and the right precentral gyrus among alcoholic patients compared to the normal controls.

**Conclusions:**

In summary, specific brain regions were identified that are associated with anger among patients with alcohol dependency.

## Background

Alcohol dependency is one of the most critical health issues in the world [1] not only because it is associated with a variety of physical health problems but also because of its association with major cerebral [2], cognitive [3], and emotional impairments [4]. Compared to cerebral and cognitive impairments, emotional deficit in alcohol dependency is studied relatively little [5, 6]. However, since the degree of appropriate social adaptation is linked with one’s ability to correctly perceive and express emotions in social interactions [7], emotional deficits experienced by alcoholics are likely to be associated with their poor social adjustments [8, 9]. Regarding the emotional reactivity among alcoholics, in fact, it is well known that alcoholics tend to experience greater negative emotions than the average population without alcohol issue [10, 11]. Using an ethological approach, Verbitskaya and colleagues [12] found less and shorter affiliative behaviors in patients (e.g., smiling, looking at the interviewer) than in the controls. This result is consistent with Dethier and Blairy’s study [6] that reported alcohol-dependent patients had exhibited fewer positive emotional facial expressions (EFEs) and more negative EFEs compared to control participants while watching a series of EFEs of different emotional categories. Likewise, studies that videotaped alcohol-dependent couples and families’ emotions during a discussion of a personally relevant issue also showed globally (verbally and nonverbally) less positive emotions and more negative emotions compared with control couples and families [13, 14].

However, the study findings on alcohol-dependents’ emotional responses have not been quite consistent, as some studies suggest that alcohol-dependent patients are likely to present an abnormal reactivity to emotional stimuli [6, 15]. For example, in Kornreich et al. [15], alcohol-dependent patients showed less cardiac responsiveness and exceedingly high or low subjective emotional responses toward emotional film excerpts, compared to control participants. On the other hand, alexithymia, another emotional impairment, is commonly found in alcoholic dependency [16]. Alexithymia was first described by Sifneos [17] and is characterized by difficulties identifying feelings and distinguishing between feelings and the bodily sensations of emotional arousal. Alcoholics may seem emotionally “flat” (i.e., they are less reactive to emotionally charged situations) [18]. Therefore, there is a need to further explore the extent of these emotional responses in alcoholism, as several affective abilities have not yet been satisfactorily evaluated.

As with the aforementioned emotional impairments, individuals suffering alcohol dependency also exhibit major cerebral impairments [2, 19]. Findings from a number of neuroimaging studies suggest that healthy brain functions associated with emotional processing, such as the amygdala, hippocampus, and anterior cingulate cortex (ACC), may have been disturbed in alcoholic patients. For example, the dysfunctions in the ACC [5] and the hippocampus and amygdala [20] are associated with the inability to accurately perceive and analyze EFEs among alcoholics. Furthermore, an abnormal reactivity in the hippocampus and parahippocampal gyrus to emotional stimuli was also found in alcohol-dependent patients [21]. It is presumable that significant dysfunctions in the neural circuitry underlying emotion processing may be related to emotional impairments in individuals with alcoholism.

This study aimed to investigate emotional and neural reactivity, specifically to an anger stimulus in alcohol-dependent patients as compared with healthy controls using functional magnetic resonance imaging (fMRI). We chose to investigate anger emotion, as anger is the most commonly expressed and least successfully handled among alcoholics [22]. Also, a higher level of anger has been reported among those with alcohol use disorder (AUD) [23, 24] in comparison with non-alcoholics, as measured by State-Trait Anger Expression Inventory (STAXI) [25]. Consistent with this notion, hostility and aggression are often observed in alcoholics [26]. However, so far, hardly any study has investigated the actual response to anger in alcohol-dependent patients in an experimental setting. We hypothesized that alcoholics may show an altered emotional response, exhibited by abnormal brain activities during an anger-induction task, notably in the orbitofrontal cortex (OFC), the ACC, and the insula, regions that are implicated in anger experience or regulation ([27–29] for a review). To test the hypothesis, neural activity was measured during a mood-induction task which consists of neutral and angry film extracts utilized in previous studies [30].

## Methods

### Participants

A total of 18 male alcohol-dependent patients (mean age 49.83 years, age range = 39–60 years) in a local inpatient alcohol treatment facility participated in this study. The final sample excluded any individuals reporting a current or history of mental disorder other than alcoholism. In consultation with their primary physician, patients on prescribed medication (i.e., sleeping pills or anti-craving medication, not psychotropic drugs) were asked to abstain from such medicines for 14 days prior to scanning.

The control group consisted of 16 alcohol-independent male volunteers with demographics similar to the alcohol-dependent group (mean age 50.06 years, age range = 31–61 years). They were recruited through research advertisements and flyers, inviting study participation. The sample excluded some individuals reporting a current or history of mental disorder, including impairment in the central nervous system. The controls refrained from drinking alcohol for at least 48 h prior to the fMRI scanning. Prior to the actual experiment, subjects received full information in the laboratory about the study, including the experimental procedure, their rights, risks and benefits, and voluntary nature of participation, and then signed the consent form. This study strictly followed the research regulations of the University Institutional Review Board for Human Subjects Research. These participants attended in another study, i.e., the emotion perception experiment conducted in our laboratory [31]. Table 1 shows the demographics and alcohol use in the patient and control groups.

**Table 1:** Demographics and alcohol use of study participants

Characteristics	Control group (*n* = 16)	Patient group (*n* = 18)	*t* value
Age (years)	50.06 (6.10)	49.83 (6.60)	0.11
Educational level	12.38 (3.57)	10.67 (4.05)	1.30
Family history (%)	0	44.4	3.56**
Number of drinks (day per week)	1.02 (1.55)	4.63 (2.25)	5.37***
Amounts of drinks (drinks per drinking day)	2.86 (2.10)	16.25 (16.08)	3.30**
Maximum number of drinks in a lifetime	8.22 (11.26)	29.77 (24.21)	3.26**
AUDIT-K	6.38 (5.54)	27.89 (9.91)	7.67***
ADS-K	28.05 (5.39)	50.00 (12.85)	6.34***

Means (standard deviations) are represented. One drink = 14 g ethanol

***P < 0.0001, **P < 0.001

### Procedure

Study participants were individually instructed on the experimental procedure prior to the experiment. Emotion-provoking film clips from movies and websites that were excerpted and validated via our previous study were used in the current study [30]. Six basic emotions were included in the study. The standardized six stimuli were employed from Sohn et al.’s [30] study. The fMRI experimental task consisted of six emotion blocks (i.e., anger, fear, disgust, sadness, happiness, and surprise) that lasted 120 s per emotion. Each emotion block was preceded by the 30-s fixation block. In the 120-s emotion block, the block was composed of a 30-s neutral condition and a 90-s emotion condition. The neutral condition was chosen from the same audio-visual clips selected for each emotional stimulus so as to match the contents, color, and hue with the emotional condition. The total scanning session took approximately 15 min. As the focus was the anger emotion in the study, only the anger condition was explored hereafter. The anger stimuli that was used for the fMRI experimental task was the audio-visual film clip where a male adult was battering a bus driver.

After the scanning session, a psychological assessment was administered; the subjects were asked to label what emotion they had felt while viewing the clip among 11 discrete emotions (i.e., happiness, sadness, anger, contempt, disgust, fear, surprise, bored, interested, neutral, and others). They also were asked to rate the intensity of the emotion on a seven-point Likert scale (1 being least angry and 7 being most angry) and report the part where they experienced the most intense emotion. The stimulus was created using an audio-visual software file which was then projected onto a screen using a mirror with subjects each wearing a headphone set to experience both visual and acoustic stimuli. Subjects’ written consent was obtained for this clinical study after the provision of a thorough explanation of the study purposes and demonstration of the procedures. The study was comprehensively reviewed and conducted in accordance with our Institutional Review Board.

### Imaging parameters

Imaging was conducted on a 3.0 T whole-body ISOL Technology FORTE scanner (ISOL Technology, Korea) equipped with whole-body gradients and a quadrature head coil. Single-shot echo planar fMRI scans were acquired in 35 continuous slices parallel to the anterior commissure-posterior commissure line. The following fMRI parameters were included: repetition time/echo time (TR/TE), 3000/30 ms; flip angle, 80; field of view (FOV), 240 mm; matrix size, 64 × 64; slice thickness, 4 mm; and in-plane resolution, 3.75 mm. So as to decrease the effect of non-steady state longitudinal magnetization, three dummy scans from the beginning of the run were excluded. T1-weighted anatomical images were obtained with a 3-D fluid-attenuated inversion recovery sequence (TR/TE = 280/14 ms, flip angle = 60, FOV = 240 mm, matrix size = 256 × 256, slice thickness = 4 mm).

### Data analysis

As the focus was the anger emotion in the study, only the anger condition was analyzed in the data analysis. For the behavioral data analysis, an independent *t* test was performed using SPSS 20.0 to compare the level of anger intensity between the alcoholic and normal control groups. In the fMRI data analysis, brain scanning data obtained during the neutral condition were compared to those with the anger condition. The imaging data were then analyzed with SPM8 (Wellcome Department of Cognitive Neurology, London, UK). Using affine transformation routines built into SPM8, all functional images were realigned with the image taken proximate for the anatomical study. The realigned scans were normalized to SPM8’s template image that uses the space defined by the Montreal Neurologic Institute, which is very similar to the Talairach and Tournoux stereotaxic atlas [32]. Sinc interpolation enabled motion correction. The functional map was smoothed with an 8-mm isotropic Gaussian kernel prior to the statistical analysis. The voxel size resulted in 2 × 2 × 2 mm^3^ from normalization. Time series data were filtered with a 240-s high-pass filter to remove any artifacts resulting from cardio-respiration and other cyclical influences.

At the first level, the data were analyzed according to a standard box-car block design, after convolving the BOLD signal with a canonical HRF as modeled in SPM8. To conduct a random effect analysis, the individual first-level analyses of the comparisons of anger condition minus neutral condition were used and created mean images for each subject. At the second level, mean images were combined with a one-sample or two-sample *t* tests to assess any group effects. In agreement with previous studies, we used a threshold of *P* < 0.001 uncorrected for the entire brain volume. An extended threshold of 20 contiguous voxels was then applied to the activation. All coordinates derived from the statistical analysis were converted from MNI to the Talairach and Tournoux stereotaxic space [32].

To extract signal changes from regions of interest (ROIs), activated clusters in ROIs were selected through xjView (http://www.alivelearn.net/xjview8/). Among the three regions showing group difference effects, we defined the right dACC as a ROI. In SPM, we created a batch file and loaded the ROI image and assigned directories where individual subjects’ files are located. We extracted signal changes from the ROI for both anger and neutral conditions in both the alcohol and control groups.

## Results

### Behavioral results

With respect to the experiment, we found that the majority of the participants stated anger as the primary emotion provoked by the experimental condition, except for two subjects in each group. In the control group, one person reported not feeling any emotion at all while the other felt surprise. In the patient group, one person reported experiencing fear and the other person feeling disgust. The anger score for the two people in each group who did not identify the anger emotion was assigned a zero (0) score. The mean intensity scores of the anger experience for the control and patient groups were 3.86 (SD 1.58) and 3.92 (SD 1.54), respectively. There was no significant difference in the experience of anger between the patient and control groups (*t* = 0.11, NS [32]).

### fMRI results

**Fig. 1:**
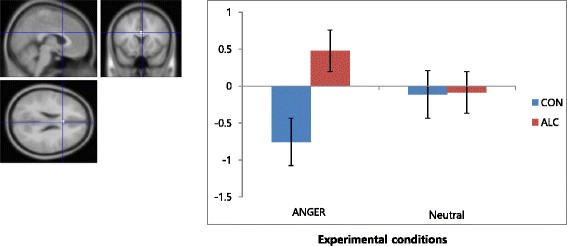
The extracted signal changes for the dACC (*above*) (average signal changes ± standard error of mean) in the control and patient groups for each experimental condition (i.e., anger and neutral conditions). Patient group > control group (anger minus neutral contrast). Parameter estimates (signal changes) extracted from the dACC averaged in each condition (i.e., anger and neutral conditions) and group (the control and patient groups), showing increases during anger condition relative to the neutral condition in the patient group. In this figure, *blue* represents the control group and *red* represents the patient group

Brain areas that were significantly activated during transient anger compared to the neutral condition were found at the level of uncorrected *P* < 0.001. In the patient group, activations were found in the left superior temporal gyrus, right middle frontal gyrus, right declive, left cuneus, right superior frontal gyrus, right inferior temporal gyrus, left superior temporal gyrus, left precentral gyrus, left declive, right middle occipital gyrus, and left inferior frontal gyrus. In the control group, activations were observed in the right superior temporal gyrus, right postcentral gyrus, right precentral gyrus, and left anterior cingulate. In the group comparison, the patient group exhibited significantly greater activity in the right precentral gyrus (BA 4), bilateral dorsal anterior cingulate gyrus (dACC), and right cuneus (BA 19) in the anger condition compared to the neutral condition at the level of uncorrected *P* < 0.001. Talairach coordinates and *t* scores of each activated area are shown in Table 2. Among the three regions showing group difference effects, we extracted signal changes in the right dACC for both groups for each experimental condition (i.e., anger and neutral conditions) as shown in Fig. 1. These graphs in the Fig. 1 show greater increases during anger condition relative to neutral condition in the patient group compared to the control group.

**Table 2:** Talairach coordinates and *t* scores of activated brain areas

Region	Side	*X*	*Y*	*Z*	BA	*t* value
Control group						
Superior temporal gyrus	Left	−32	12	−32	38	5.87
Middle frontal gyrus	Right	42	26	44	8	4.45
Declive	Right	38	−70	−16		4.40
Cuneus	Left	−18		−106	18	4.39
Superior frontal gyrus	Right	16	48	40	8	4.16
Inferior temporal gyrus	Right	52	−10	−22	20	3.89
Superior temporal gyrus	Left	−56	−48	18	22	3.78
Precentral gyrus	Left	−32	−16	60	6	3.73
Declive	Left	−46	−66	−18		3.73
Inferior temporal gyrus	Right	56	−72	0		3.56
Middle occipital gyrus	Right	36	−82	4	19	3.54
Inferior frontal gyrus	Left	−40	28	2	47	3.51
		−18	−60	−50		3.43
Alcohol group						
Superior temporal gyrus	Right	46	−10	−6	22	3.70
	Right	46	2	−14		3.32
Postcentral gyrus	Right	34	−20	30		3.25
Precentral gyrus	Right	28	−22	70	4	3.12
Anterior cingulate	Left	−1	13	22	24	3.08
Control group < alcohol group						
Precentral gyrus	Right	28	−24	72	4	3.19
Anterior cingulate	Left	0	14	24	24	3.10
Cuneus	Right	14	−86	38	19	2.98

Brain activation comparisons between the anger condition and the neutral condition in each group and contrasting effects between two groups (i.e., the patient group versus control group) (uncorrected *P* < 0.001)

## Discussion

The aim of this study was to provide an understanding of the underlying psychological and neural mechanisms of transient anger among individuals with alcoholism. This is the first study to find alcoholics’ psychological or neural response during anger-induction paradigm. Beforehand, areas that were significantly activated in both the control and alcoholic groups were consistent with the previous research outcomes on the brain regions responsible for anger processing. Specifically, they are the ACC and orbital frontal gyrus [27, 33].

Interesting findings of the study are that the dACC activity was decreased in the control group and increased in the alcoholic group despite the comparable level of subjective anger to standardized anger-induction stimuli. The greater dACC activation in the alcohol group relative to the control group is consistent with Park et al. [31] in the evaluation of angry faces and feeling anger in the individuals with AUD college students [34]. The dACC is activated when participants are angered [28] and seems to be implicated in anger control [35]. The role of the dACC in social-affective contexts is that it could be involved in producing feelings that is linked to the intensity of a number of emotions that are involved in negative social situations such as anger provocation [36–38]. Patients with alcohol dependency in the study appear to experience a higher level of negative feelings when they were exposed to anger induced by audio-visual film clips. This is consistent with the notion that alcoholics are hostile and aggressive [26, 39]. However, even though there was significant difference between the two groups in the dACC activation, there was not any difference in self-reported anger experience between the AUD group and the control group. It is plausible that brain activation might be a more sensitive measure compared to the behavior measure via a self-report. For example, in the study of Salloum et al. [5], alcoholics showed significantly less activation in the ACC than the healthy controls while both groups did not differ in accurately identifying the intensity level on the simple emotional-decoding task. In another study, the high depressive group showed greater regional activity in relation to the low depressive group in the brain regions known to be involved in sadness, notably the insula, ACC, and caudate nucleus, even though the groups did not differ in the current mood [40]. These previous findings suggest that the brain activity via an fMRI can be more sensitive compared to the behavioral measure for the detection of the human mind or behavior.

This study has a few limitations. Firstly, in the further study, an additional measure might be needed to confirm the difference in the anger experience among alcoholics. Despite our findings showing an abnormality in brain function for patients with alcohol dependency in response to anger stimuli, they did not differ from the control group in terms of behavioral outcomes on the level of anger intensity. Even though we provided plausible explanation for the contrasting results between brain activity and behavioral measure, there are other possibilities for the result. One is that self-reports might not be a reliable measure to detect alcoholics’ emotional or cognitive processes. Philippot et al. showed that alcoholics reported equivalent degrees of difficulty even though they performed poorly in the emotional-decoding tasks than normal controls [41]. Secondly, the period of alcohol abstinence varied across study participants (from 11 to 2051 days), which made it difficult to suggest a relationship between the length of abstinence and their anger experience. Despite the limitation, these findings suggest specific brain regions associated with anger processing among patients with alcohol dependency.

## References

[1] Harper C, Matsumoto I (2005). Ethanol and brain damage. Curr Opin Pharmacol.

[2] Harper C (2007). The neurotoxicity of alcohol. Hum Exp Toxicol.

[3] Pitel AL, Beaunieux H, Witkowski T (2007). Genuine episodic memory deficits and executive dysfunctions in alcoholic subjects early in abstinence. Alcohol Clin Exp Res.

[4] Dethier M, El Hawa M, Duchateau R (2014). Emotional facial expression recognition and expressivity in type I and type II alcohol dependent patients. J Nonverbal Behav.

[5] Salloum J, Ramchandani VA, Bodurka J (2007). Blunted rostral anterior cingulate response during a simplified decoding task of negative emotional facial expressions in alcoholic patients. Alcohol Clin Exp Res.

[6] Dethier M, Blairy S (2012). Capacity for cognitive and emotional empathy in alcohol-dependent patients. Psychol Addict Behav.

[7] Feldman RS, Philippot P, Custrini RJ, Feldman RS (1991). Social competence and nonverbal behavior. Fundamentals of non-verbal behavior.

[8] Maurage P, Campanella S, Philippot P (2009). Impaired emotional facial expression decoding in alcoholism is also present for emotional prosody and body postures. Alcohol Alcohol.

[9] Uekermann J, Channon S, Winkel K (2007). Theory of mind, humour processing and executive functioning in alcoholism. Addiction.

[10] Elkins IJ, King SM, McGue M (2006). Personality traits and the development of nicotine, alcohol, and illicit drug disorders: prospective links from adolescence to young adulthood. J Abnorm Psychol.

[11] Sher KJ, Trull TJ, Bartholow BD, Leonard KE, Blane HT (1999). Personality and alcoholism: issues, methods, and etiological processes. Psychological theories of drinking and alcoholism.

[12] Verbitskaya EV, Krupitsky EM, Burakov A (2007). Nonverbal behavior of human addicts: multimetric analysis. Addict Behav.

[13] Jacob T, Leonard K (1992). Sequential analysis of marital interactions involving alcoholic, depressed, and nondistressed men. J Abnorm Psychol.

[14] Jacob T, Leonard KE, Haber JR (2001). Family interactions of alcoholics as related to alcoholism type and drinking condition. Alcohol Clin Exp Res.

[15] Kornreich C, Philippot P, Verpoorten C (1998). Alcoholism and emotional reactivity: more heterogeneous film-induced emotional response in newly detoxified alcoholics compared to controls—a preliminary study. Addict Behav.

[16] Taieb O, Corcos M, Loas G (2002). Alexithymia and alcohol dependence. Ann Med Interne (Paris).

[17] Sifneos PE (1973). The prevalence of ‘alexithymic’ characteristics in psychosomatic patients. Psychother Psychosom.

[18] Oscar-Berman M, Marinkovic K (2003). Alcoholism and the brain: an overview. Alcohol Res Health.

[19] Fitzpatrick LE, Jackson M, Crowe SF (2008). The relationship between alcoholic cerebellar degeneration and cognitive and emotional functioning. Neurosci Biobehav Rev.

[20] Marinkovic K, Oscar-Berman M, Urban T (2009). Alcoholism and dampened temporal limbic activation to emotional faces. Alcohol Clin Exp Res.

[21] Gilman JM, Hommer DW (2008). Modulation of brain response to emotional images by alcohol cues in alcohol-dependent patients. Addict Biol.

[22] Han KW, Kim MJ, Kim SG (1996). Study of the conditioned stimuli provoking alcohol craving in the patients with alcohol dependence. J Korean Neuropsychiatr Assoc.

[23] Walfish S, Massey R, Krone A (1990). MMPI profiles of adolescent substance abusers in treatment. Adolescence.

[24] Potter-Efron PS, Potter-Efron RT (1991). Anger as a treatment concern with alcoholics and affected family members. Alcohol Treat Q.

[25] Spielberger CD, Krasner SS, Soloman EP, Janisse MP (1988). The experience, expression and control of anger. Health psychology: individual differences and stress.

[26] Handelsman L, Stein JA, Bernstein DP (2000). A latent variable analysis of coexisting emotional deficits in substance abusers: alexithymia, hostility, and PTSD. Addict Behav.

[27] Dougherty DD, Shin LM, Alpert NM, Pitman (1999). Anger in healthy men: a PET study using script-driven imagery. Biol Psychiatry.

[28] Denson TF, Pedersen WC, Ronquillo J (2009). The angry brain: neural correlates of anger, angry rumination, and aggressive personality. J Cogn Neurosci.

[29] Lindquist KA, Wager TD, Kober H (2012). The brain basis of emotion: a meta-analytic review. Behav Brain Sci.

[30] Sohn J-H, Lee O-Y, Suk J-A, Park, et al. Development and validation of emotion-inducing film stimuli. Seoul: Korean Federation of Science and Technology Societies; 2005.

[31] Park M-S, Kim S-H, Sohn S (2015). Brain activation during processing of angry facial expressions in patients with alcohol dependency. J Physiol Anthropol.

[32] Talairach J, Tournoux P (1988). Co-planar stereotaxic atlas of the human brain.

[33] Damasio AR, Grabowski TJ, Bechara A, Damasio H, Ponto LL, Parvizi J, Hichwa RD (2000). Subcortical and cortical brain activity during the feeling of self-generated emotions. Nat Neurosci.

[34] Park M-S, Sohn S, Seok J-W (2015). Altered patterns of brain activity during transient anger among young males with alcohol use disorders: a preliminary study. Korean Soc Emot Sensibility.

[35] Denson TF, Dobson-Stone C, Ronay R, von Hippel W, Schira MM (2014). A functional polymorphism of the MAOA gene is associated with neural responses to induced anger control. J Cogn Neurosci.

[36] Eisenberger NI, Lieberman MD, Williams KD (2003). Does rejection hurt? An fMRI study of social exclusion. Science.

[37] Eisenberger NI, Way BM, Taylor SE, Welch WT, Lieberman MD (2007). Understanding genetic risk for aggression: clues from the brain’s response to social exclusion. Biol Psychiatry.

[38] Kross E, Egner T, Ochsner K, Hirsch J, Downey G (2007). Neural dynamics of rejection sensitivity. J Cogn Neurosci.

[39] Tivis LJ, Parsons OA, Nixon SJ (1998). Anger in an inpatient treatment sample of chronic alcoholics. Alcohol Clin Exp Res.

[40] Lévesque ML, Beauregard M, Ottenhof KW, Fortier É, Tremblay RE, Brendgen M, Boivin M (2011). Altered patterns of brain activity during transient sadness in children at familial risk for major depression. J Affect Disord.

[41] Philippot P, Kornreich C, Blairy S, Baert I, Dulk AD, Bon OL, Verbanck P (1999). Alcoholics’ deficits in the decoding of emotional facial expression. Alcohol Clin Exp Res.

